# Prosocial behaviours under collective quarantine conditions. A latent class analysis study during the 2020 COVID‐19 lockdown in Italy

**DOI:** 10.1002/casp.2571

**Published:** 2021-09-18

**Authors:** Giovanni Aresi, Fortuna Procentese, Silvia Gattino, Iana Tzankova, Flora Gatti, Christian Compare, Daniela Marzana, Terri Mannarini, Angela Fedi, Elena Marta, Antonella Guarino

**Affiliations:** ^1^ Psychology Department Università Cattolica del Sacro Cuore, Largo Gemelli Milan Italy; ^2^ CERISVICO Research Centre on Community Development and Organisational Quality of Life Brescia Italy; ^3^ Department of Humanities University of Naples Federico II Naples Italy; ^4^ Department of Psychology University of Torino Torino Italy; ^5^ Department of Psychology University of Bologna Cesena Italy; ^6^ Department of History Society and Human Studies University of Salento Lecce Italy

**Keywords:** community, COVID‐19, lockdown, person‐centred approach, prosocial behaviours

## Abstract

We aimed to identify the patterns of prosocial behaviours under collective quarantine conditions. Survey data were collected from a sample of Italian adults during the March May 2020 COVID‐19 lockdown in Italy. Participants reported on offline and online prosocial behaviours, sense of community responsibility (SoC‐R) and perceptions of community resilience. Latent class analysis (LCA) was used for data analysis. A total of 4,045 participants completed the survey, and 2,562 were eligible (72% female; mean age 38.7 years). LCA revealed four classes of prosocial behaviours: *Money donors* (7%), *Online and offline helpers* (59%), *Online health information sharers* (21%) and *Neighbour helpers* (13%). The classes were partially invariant across age groups (18‐35 and 35‐65 years). Being a man, having achieved a higher educational level and higher SoC‐R scores were associated with belonging to the *Online and offline helper* class. The members of this class also reported the greatest perceptions of community resilience. The results provide insight on the multidimensionality of prosociality under collective quarantine conditions. *Online and offline helpers* could be targeted for promoting sustained altruism and involvement in community organisations. For the other groups, programmes should aim at eliminating barriers to help others in multiple ways. Please refer to the Supplementary Material section to find this article's Community and Social Impact Statement.

## 1 INTRODUCTION

The expression ‘catastrophe compassion’ was used to describe how people react to large‐scale disasters by engaging in altruistic behaviour (Zaki, 2020). Scholars suggested that these forms of prosociality during collective tragic events arise from shared social identities and emotional connection with other people who are facing the same hardships (Drury, 2018; Zaki, 2020). Visibly displaying prosocial and selfless acts can also foster other people's in‐group commitment and prompt them to act accordingly (Van Bavel et al., 2020).

In the first months of 2020, the world was hit by the SARS‐CoV‐2 pandemic and countries all over the world imposed quarantine measures to reduce the spread of the virus. First in Europe, Italy imposed a strict lockdown starting on 8 March that was partially eased on 4 May. These measures proved effective against the spread of the virus, but caused disruption in social and community life (Brooks et al., 2020). Despite the difficulties, millions of people reacted by engaging in a variety of altruistic behaviours, such as volunteering, donating money, and offering online social and emotional support to others (Brooks et al., 2020).

By using data on prosocial behaviours from a large sample of Italian adults, this study will examine the specifics of how prosociality was expressed during the March–May 2020 COVID‐19 lockdown when face‐to‐face activities were strongly limited by restrictions imposed by the authorities.

Prosocial behaviours have been distinguished according to different dimensions (e.g., spontaneous informal vs. planned formal; personal vs. impersonal), as well as the amount of effort required from the helper (Coyne et al., 2018; Padilla‐Walker & Carlo, 2014). Low‐cost behaviours are relatively easy and often one‐off actions of helping and kindness, such as sending an uplifting message. High‐cost behaviours, such as volunteering in emergency situations, require prolonged engagement and moral courage, and may be against one's own interests (Eisenberg & Spinrad, 2014; Niesta Kayser, Greitemeyer, Fischer, & Frey, 2010).

The results of research offer insight into the likelihood and motivations of prosocial behaviour in emergency situations (Fischer, Greitemeyer, Pollozek, & Frey, 2006; Rand & Epstein, 2014; Rodríguez, Trainor, & Quarantelli, 2006), although the COVID‐19 pandemic represents a massive global health crisis and the measures to contain it were unprecedented. For this reason, this crisis is somehow unique and required significant shifts in behaviour (including prosocial behaviour) (Van Bavel et al., 2020). People were forced home for a long period, and how prosociality was expressed had to be adapted. Little is known, however, in regard to the use of online forms of prosocial behaviour during sanitary crises (e.g., Palen, Hiltz, & Liu, 2007) and whether they coexist with offline behaviours. Online and offline altruistic conducts share fundamental characteristics and beneficial consequences for the receiver, the giver and the community overall (Sproull, Conley, & Moon, 2013; Wright & Pendergrass, 2016). The Internet and social networks provide additional opportunities for people to help, especially those who are confined due to geographical or other resource limitations; they can provide means for sharing information, virtual communication and learning from others' personal experience and knowledge in preparation for future events (Palen et al., 2007). This may be of particular importance when collective quarantine measures are enforced and action is mostly confined to the digital sphere. Some have indeed suggested that digital platforms played a key role in mitigating the effects of the COVID‐19 pandemic (Miao, Schwarz, & Schwarz, 2021).

Research on the multidimensionality of prosocial behaviour stresses the importance of considering different types of behaviour simultaneously. This can be achieved using the person‐centred approach (i.e., latent class analysis or LCA), which relaxes ‘*the assumption that all individuals are drawn from a single population, and consider the possibility that the sample might include multiple subpopulations characterized by different sets of parameters’* (Morin, Bujacz, & Gagné, 2018, p. 805). This results in a classification system that groups individuals into distinct profiles or classes. The innovation of this approach is to use a bottom‐up approach, first identifying classes of individuals and then studying variations between these behavioural patterns across socio‐demographic and psychosocial factors.

### 1.1 Predictors of prosocial behaviours under emergency conditions

Little is known as to whether the patterns of prosocial behaviours during emergencies and collective quarantine may differ across socio‐demographic characteristics. First, there is evidence that gender may be linked to different prosocial orientations in conformity with gender role expectations (Eagly, 2009). Studies suggest that—both among adolescents and adults—women are more prosocial and altruistic than men (Rand, Brescoll, Everett, Capraro, & Barcelo, 2016; Xiao, Hashi, Korous, & Eisenberg, 2019) and are more inclined than men to be involved in forms of civic participation, such as voluntary work or care work (Cicognani, Zani, Fournier, Gavray, & Born, 2012; Malin, Tirri, & Liauw, 2015; Wilson, 2000). Nevertheless, some prosocial behaviours, such as helping strangers in situations that require taking the initiative or where others are present, are more frequent among men (i.e., heroic and public behaviour) (Diekman & Clark, 2015; Eagly, 2009; Xiao et al., 2019). For these reasons, men may be more active during emergencies because they generally engage more than women in prosocial behaviour that involves real or perceived physical risk and their behaviours are more agentic and collectively oriented than women's (Eagly, 2009; Espinosa & Kovářík, 2015). In terms of age differences, prosocial behaviours are the lowest during young adulthood because of the instability in life and relationships, a greater focalisation on oneself and one's educational and work goals (Eisenberg, Cumberland, Guthrie, Murphy, & Shepard, 2005; Freund & Blanchard‐Fields, 2014), and increase as individuals achieve a more stable role in society in later adulthood and old age, greater empathy and the adoption of generative goals. Young people, however, may have already been familiar with the digital world before the COVID‐19 health emergency and therefore have been more prone to help online (Xie et al., 2020). In regard to socio‐cultural and economic status, there is evidence that engagement in volunteerism is greater among those with higher income and educational level (Independent Sector, 2001). This is likely to be related to greater empathy towards others and awareness of the problems they face, coupled with greater expectancy of effectiveness of one's actions (Wilson, 2000).

From a psychosocial perspective, prosocial behaviours have been explained using constructs revolving around helpers' sense of responsibility for others (Yang et al., 2020). At a community level, responsibility towards others has been understood as sense of community responsibility (SoC‐R), which refers to the feeling of responsibility towards other community members and the community as a whole, that enhances individuals' motivation to help (Nowell & Boyd, 2010; Nowell & Boyd, 2014).

### 1.2 The relationship between prosociality and community resilience

Research has demonstrated the benefits of prosociality in the face of disasters and emergencies, including during a pandemic (Varma, Chen, Lin, Aknin, & Hu, 2020). Helping others contributes to enhancing helpers' physical and psychological well‐being (Curry et al., 2018; Dunn, Whillans, Norton, & Aknin, 2020; Pozzi, Marta, Marzana, Gozzoli, & Ruggieri, 2014), even when beneficiaries are distant and not physically present (Martela & Ryan, 2016). Altruistic behaviours also lead to positive collective outcomes, such as an increase in opportunities for social relationships, solidarity, reciprocal support and feelings of being a competent individual and community member (Drury, 2018; Vezzali, Drury, Versari, & Cadamuro, 2016). These are critical elements upon which community resilience is built (Heid, Christman, Pruchno, Cartwright, & Wilson‐Genderson, 2016; Norris, Stevens, Pfefferbaum, Wyche, & Pfefferbaum, 2008). Therefore, the adoption of prosocial behaviours by community members is also supposed to foster greater perceptions of the community's ability to cope under difficult circumstances (i.e., its resilience or capability to respond to negative collective events and stressors) (Magis, 2010). During the COVID‐19 pandemic, prosocial behaviours may have strengthened resilience by adding a sense of purpose and adaptive meaning in surviving the crisis. Strong communities are necessary to provide material and emotional resources when individuals need them the most. Indeed, prosocial acts of tolerance, support and kindness can have a buffering function against the negative effects of an emergency (PeConga et al., 2020). Community resilience includes different dimensions (Pfefferbaum, Pfefferbaum, Nitiéma, Houston, & Van Horn, 2014), although two are likely to be important outcomes of altruistic behaviours: the first one being community transformative potential (i.e. perception of the ability to analyse and understand collective experiences in order to assess and build community skills to face them) and the second being the perception of the community's capacity to cope with disasters (i.e. community readiness and recovery in the face of disasters).

## 2 AIMS

We used LCA to examine data on prosocial behaviours among Italian adults (18–65 years) during the March May 2020 COVID‐19 national lockdown in Italy. From the very early days of the health emergency, people over the age of 65 years were encouraged by institutional communication to self‐isolate and were also exempted from any voluntary work outside their home with implications on their prosocial behavioural patterns. For these reasons, this age group was excluded from our analyses.

Our study aimed to (a) identifying distinct subgroups (i.e., classes) of individuals based on a set of prosocial behaviours and (b) examining the consistency of the latent class solution across age groups. Because the transition to adulthood seems to be postponed until the age of 35 years among Italians when compared to other countries (Scabini, Marta, & Lanz, 2006), two groups of young adults and older adults (18–35 and 36–65 years) will be compared; (c) examine whether gender, level of education and community sense of responsibility distinguish between the classes; and (d) whether differences exist across classes in perceptions of community resilience.

Because there is no research using LCA on the patterns of prosocial behaviours during emergencies, we found it difficult to make specific hypotheses. However, we did expect young adults to belong to profiles that were more active online, but adults aged over 35 years to engage in a greater variety of altruistic behaviours, both online and offline. In regard to our third and fourth research questions, we expected that being a man and having a greater sense of responsibility towards their community would be associated with belonging to profile(s) that are characterised by greater involvement in a variety of—including high cost—prosocial behaviours. This latter profile(s) should also display greater perceptions of community resilience.

## 3 METHODS

### 3.1 Study design

This study was the result of a collaborative effort by five Italian universities: the University of Bologna, University of Naples Federico II, University of Torino, University of Salento and Università Cattolica del Sacro Cuore. Because of the nationwide lockdown restrictions, each university research team recruited participants through direct contact via email or social networks (e.g., Facebook) in their respective geographic areas. Participants of all ages, except for people under the age of 18 years, were emailed a link to a survey. To increase reach, local municipalities and community organisations were involved and a snowball sampling technique was used whereby respondents were asked to forward the link to people they knew. Respondents did not receive any incentive for their participation. Data were collected between 12 April and 21 May 2020. Ethical approval was obtained from the Human Research Ethics Committee at the Università degli Studi di Bologna for all aspects of the current research. All participants provided informed consent for taking part in the study. Given the importance of quickly collecting data during an unprecedented time, our study was not pre‐registered and results should be considered exploratory.

### 3.2 Measures

The survey included data on age, gender, level of education (from 1 = *lower than secondary school* to 6 = *post‐tertiary degree*), occupation, province of residence, measures of prosocial behaviours, SoC‐R and community resilience.

**Prosocial behaviours**. Eight dichotomous (No = 0, Yes = 1) items assessing engagement in a variety of online and offline behaviours were adapted from existing scales to the specifics of the lockdown (Enchikova et al., 2019; Rushton, Chrisjohn, & Fekken, 1981). Respondents were asked to answer the question ‘*Since the beginning of the COVID‐19 emergency, have you engaged in any of the following behaviours?*’. The items were the following: *‘I have worked in a volunteer association for practical help, such as transport, delivery of drugs’* (Volunteered); *‘I have donated money to a hospital/ health service’* (Donated money); *‘I have helped a neighbour’* (Helped a neighbour); *‘I have given classes to share my competences (soft skills and professional) with others’* (Shared competencies online); *‘I have shared verified and official health advice on social networks’* (Shared health advice online); *‘I have offered school services for children/teenagers at home’* (Helped school children online); *‘I have posted messages of hope on social networks’* (Created hope content online); and *‘I have created a sharing platform on the Web’* (Created an online sharing platform). The items represented both low‐cost (e.g., posting messages of hope online),high‐cost (e.g., volunteering for associations), offline personal (e.g., helping neighbours) and online impersonal (e.g., sharing advice online) behaviours.

**Sense of community responsibility**. We used the Italian version of the SoC‐R scale (Prati et al., 2020). The scale consists of six items (e.g., ‘*It is easy for me to put aside my own agenda in favour of the greater good of my community*’*)*. The answers were scored from 1 (*completely disagree*) to 5 (*completely agree*).

**Community resilience.** We used two subscales of the Community Advancing Resilience Toolkit (CART) (Pfefferbaum et al., 2014): Transformative potential (three items; e.g., ‘*My community looks at its successes and failures so it can learn from the past*’) and Disaster management (four items; e.g., ‘*My community can provide emergency services during a disaster*’). The answers were scored from 1 (*completely disagree*) to 5 (*completely agree*).

### 3.3 Data analysis

Based on the eight dichotomous indicators, the subgroups of individuals characterised by the common patterns of multiple prosocial behaviours were identified using LCA. Thus, analyses were restricted to those who engaged in these conducts (sensitivity analyses also demonstrated the instability of the model if non‐prosocial participants were included). Following established recommendations (Lanza, Dziak, Huang, Xu, & Collins, 2011), a series of statistical models were estimated in the overall sample, followed by the examination of measurement invariance across 18–35‐ and 36–65‐year age groups. For a description of the statistical (absolute and relative model fit indices) and conceptual standards used to compare the different profile solutions, see Sorgente, Lanz, Serido, Tagliabue, and Shim (2019). The three‐step procedure (Asparouhov & Muthén, 2014) was used to test the effect on class membership probabilities of gender, educational level and SoC‐R. In the final set of analyses, we included the two Transformative potential and Disaster management CART subscales as outcome variables and estimated their mean for each of the four latent classes by age group. Analyses were performed in MPlus 7 (Muthén & Muthén, 1998‐2015) using the robust maximum‐likelihood estimator.

## 4 RESULTS

### 4.1 Participants

A total of 4,045 participants completed the survey. Those who completed the survey after the national lockdown in Italy was eased on 4 May 2020, as well as who did not report their age (*N* = 2) and those aged 65 years and above (*N* = 154), were excluded (*N* = 793). Those who reported to have performed at least one prosocial behaviour were more likely to be women (*χ2* [1] = 5.996, *p* < .05); 84 and 80 % of women and men, respectively, reported at least one behaviour.

In LCA analyses, only participants who reported at least one prosocial behaviour and had no missing values on one or more indicators or predictor variables were included (*N* = 2,562). Slightly more than two‐thirds (71.9%, *N* = 1,842) of participants were female, and the mean age was 38.7 years (*SD* = 12.88; range 18–65). Nearly half of the sample (46.9%, *N* = 1,201) were 35 years of age or younger, whereas 53.1% (*N* = 1,361) were 36 years of age or older. The participants' occupation status was distributed as follows: 16.7% students, 72.1% workers, 8.3% unemployed and 18.2% retired. In terms of geographic area, 70.3% lived in the North and 29.7% in the Central and South Italy.

### 4.2 Descriptive statistics

Table 1 displays the proportion of participants who reported each prosocial behaviour by gender and age group, and for the whole sample. Men were more likely to report having volunteered, whereas women were more likely to report having helped a neighbour, helped school children online and created hope content online. Participants over the age of 35 years were more likely to report having helped a neighbour and to have shared verified health information via social networks. No age differences were observed with respect to the other behaviours. Table 2 reports on descriptive statistics and correlations between continuous predictor and outcome variables. The internal consistency of all scales included as covariates or outcomes was within conventional limits, varying from α = 0.79 to α = 0.89.

**TABLE 1:** Proportion of respondents reporting prosocial behaviours, by gender and age group

Prosocial behaviour	Overall (*N* = 2,562)	Gender			Age		
		Women (*N* = 1,842)	Men (*N* = 720)	χ2	18–35 years (*N* = 1,201)	36–65 years (*N* = 1,361)	Chi‐square test
Volunteered	8.8	8.1	10.6	3.745*	8.9	8.7	0.022
Donated money	35.1	34.5	36.5	0.909	35.6	34.7	0.214
Helped a neighbour	48.3	49.8	44.4	5.908**	43.5	52.5	21.024***
Shared competencies online	19.1	18.3	21.0	2.305	17.8	20.2	2.354
Shared health advice online	53.2	54.0	51.4	1.378	47.6	58.2	28.606***
Helped school children online	13.2	15.2	8.2	22.134***	13.6	12.9	0.228
Created hope content online	34.7	36.0	31.3	5.259*	33.4	35.9	1.714
Created an online sharing platform	19.1	18.4	21.0	2.208	17.5	20.6	3.932*

*Note*: Values indicate % reporting the behaviour, *N* = sample size.

* *p* < .05.

** *p* < .01.

*** *p* < .001.

**TABLE 2:** Descriptive statistics and correlations between continuous predictor and outcome variables

Variable	*M*	*SD*	1	2	3	4	5
1. Age (years)	38.7	12.88	‐				
2. Level of education	3.2	1.50	0.024	‐			
3. SoC‐R	3.7	0.67	0.097***	−0.049*	‐		
4. CART—Transformative potential	3.6	0.83	0.033	−0.042*	0.275***	‐	
5. CART—Disaster management	3.5	0.82	0.050*	−0.079*	0.290***	0.692***	‐

*Note*: *N* = 2,562.

Abbreviations: CART, Community Advancing Resilience Toolkit; SoC‐R, sense of community responsibility.

* *p* > .05.

** *p* > .01.

*** *p* > .001.

### 4.3 Identification of latent classes of prosocial behaviours

We compared models with two to seven latent classes. However, the seven‐class model did not converge and was not reported. As seen in Table 3, although each of the relative fit indices (CAIC and ssBIC) decreased with each additional solution, the relative reduction in these values substantially diminished beyond the four‐class solution. For example, the difference in aBIC between the three‐class solution and the four‐class solution was 99.592 (22,019.096–21,919.504), whereas this difference was only 36.775 (21,919.504–21,882.729) when comparing the four‐class and five‐class solutions. The other fit indices did not provide clear evidence to support either the four‐ or five‐class model, except that the five‐class solution exhibited the highest value of cmP, and the four‐class model presented a number of standardised residual larger than |3| just above the 5% threshold (Stdres = 5.24%). For these reasons, the five‐model solution was examined first. Inspection of item probabilities of this model, however, revealed that latent classes were not clearly distinguished (Table S1). On the other hand, the four‐class model classes were relatively distinguishable and interpretable. Thus, this model was deemed the best‐fitting, most interpretable and most parsimonious solution to the data. For this model, entropy was above acceptability thresholds (> 0.70).

**TABLE 3:** Model fit statistics for latent class analysis models with two to six latent classes

Model	*‐LL*	SCF	χ2 *LRT* (*p* value)	Stdres	LMR‐LRT (*p* value)	BLRT	CAIC	ssBIC	BF	cmP	SSS	Entropy
Two‐class	−11,028.624	1.15	1,112.953 (*p* < .001)	7.33%	0.000	0.000	22,091.248	22,136.659	0.00	0.00	769	0.557
Three‐class	−10,948.821	1.21	535.102 (*p* < .001)	3.66%	0.000	0.000	21,949.643	22,019.096	0.00	0.00	374	0.440
Four‐class	−10,878.005	1.01	544.122 (*p* < .001)	5.24%	0.000	0.000	21,826.009	21,919.504	0.02	0.02	172	0.715
Five‐class	−10,838.596	1.07	405.495 (*p* < .001)	3.14%	0.000	0.000	21,765.193	21,882.729	0.00	0.98	243	0.717
Six‐class	−10,797.298	1.04	325.472 (*p* < .001)	1.05%	0.000	0.000	21,700.596	21,842.173	‐	388.93	209	0.743

*Note*: BF, Bayesian factor; BLRT, bootstrapped likelihood ratio test; CAIC, consistent Akaike information criterion; cmP, approximate correct model probability; LL, log likelihood; Stdres, standardised residuals; LMR‐LRT, Lo–Mendell–Rubin likelihood ratio test; SCF, scaling correction factor of the robust maximum‐likelihood estimator; ssBIC, sample size adjusted Bayesian information criterion; SSS, smaller class numerosity; χ2 LRT, likelihood ratio chi square goodness‐of‐fit.

Table 4 presents the results of the selected four‐class model. The numbers below each subgroup heading (item‐response probabilities) represent the likelihood that the participants in each latent class reported exhibiting a specific prosocial behaviour. About 7% of the sample belonged to the ‘*Money donor’* class, defined by very low probabilities of reporting any of the prosocial behaviours except donating money to a hospital or healthcare provider. Response probability to this indicator was 1.00, reflecting certainty of this behaviour. In contrast, ‘*Online and offline helpers’* (59.1% of the sample) were likely to report a variety of both offline (e.g., helping a neighbour) and online (e.g., sharing verified information via social networks) prosocial behaviours, although none were dominant or reflected high certainty (the greatest being 0.54). Interestingly, among all classes, members of this class showed the greatest probability of having been actively engaged in a volunteer organisation providing practical help during the lockdown. About 20% of the sample was classified as *‘Online health information sharers’*, who were distinguished by elevated probabilities of sharing verified health information via social networks. ‘*Neighbour helpers’* (13% of the sample) were characterised by high probabilities of helping neighbours only.

**TABLE 4:** Item‐response probabilities and class prevalence rates for four‐class LCA model for the full sample

	Latent class			
	Money donors	Online and offline helpers	Online health information sharers	Neighbour helpers
Volunteered	0.04	0.12	0.02	0.07
Donated money	**1.00**	0.31	0.28	0.31
Helped a neighbour	0.00	0.48	0.32	**1.00**
Shared competencies online	0.06	0.31	0.00	0.04
Shared health advice online	0.10	**0.54**	**1.00**	0.00
Helped school children online	0.06	0.21	0.00	0.05
Created hope content online	0.03	0.46	0.36	0.00
Created an online sharing platform	0.03	0.30	0.03	0.06
**Estimated prevalence**	7.2%	59.1%	20.5%	13.3%
	**Means of CART outcomes**			
Transformative potential	3.53	3.78	3.12	3.44
Disaster management	3.50	3.61	3.23	3.44

*Note*: Entries in bold font indicate class‐defining probabilities (>0.50).

### 4.4 Assessing invariance of it**e**m‐response probabilities across age groups

**FIGURE 1:**
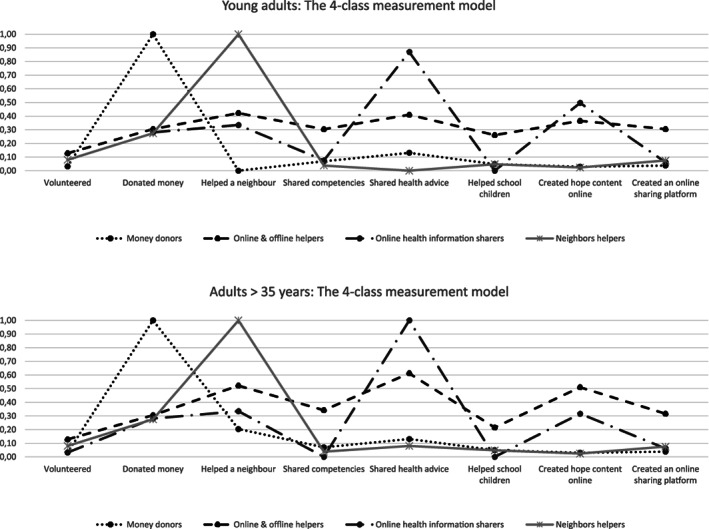
Item‐probabilities plots for the partial invariant four‐class model by age group

As shown in Table 5, the full invariant model (in which all parameters were kept equal across age groups) was statistically different from the baseline model, that is, the model in which all parameters were free to vary (*p* < .001). Therefore, it was not possible to assume full measurement invariance. We had to free (i.e., let them vary across age groups) 11 parameters before obtaining a model statistically equal to the baseline (*p* > .05). Parameters were let free one at a time starting from the greatest deviation in absolute value between the baseline and the full invariance models. The majority of the *Online and offline helper* class' item‐response probabilities (indicators were *Helped a neighbour*, *Shared competencies online*, *Shared health advice online*, *Helped school children online*, *Created hope content online*, *Created an online sharing platform*) were free to differ across the two groups. All free parameters, except one (*Helped school children online*), showed an increased probability among older adults as compared to young adults. Despite the differences in those 11 parameters, the interpretation of the *Online and offline helpers* and the other classes remained broadly the same across age groups. The item‐response probability plots of the partially invariant solution are reported in Figure 1.

**TABLE 5:** Chi‐square difference tests based on log likelihood values

	‐LL	SCF	*d*	Δ	*df*	*p*‐value
Baseline model	−12,588.82	1.07	71			
Full invariance	−12,642.00	1.01	39	94.09	32	<.001
Partial invariance	−12,606.01	1.03	50	30.08	21	.090

*Note*: ‐LL, model log likelihood; SCF, scaling correction factor of the robust maximum‐likelihood estimator; *d*, number of free parameters; Δ, difference test value; *df*, degree of freedom of the difference test.

### 4.5 Predictors of latent class membership

Gender, educational level and SoC‐R were included in the model to test their impact on class membership, separately by the age group (Table 6). The *Online and offline helper* class was selected as the reference category. The associations between latent class membership and the covariates show that men were about 40 to 50% less likely to belong to any class compared to the *Online and offline helpers* (OR range 0.34 0.59), except for the *Neighbour helper* class among adults over 35 years. Similarly, a lower level of education achieved was associated with a lower chance to belong to any class when compared to *Online and offline helpers*, except for young adult *Money givers*. Lastly, lower SoC‐R scores were related to increased probabilities of being a member of any class in both samples compared to the *Online and offline helper* class.

**TABLE 6:** Associations between latent class membership, gender, level of education and sense of community responsibility (SoC‐R) by age group

	Young adults			Adults 35─65 years		
	Class money givers OR (95% CI)^**^	Class *Online health information sharers* OR (95% CI)^**^	Class *Neighbours helpers* OR (95% CI)^**^	Class *Money donors* OR (95% CI)^**^	Class *Online health information sharers* OR (95% CI)^**^	Class *Neighbours helpers* OR (95% CI)^**^
Gender (male)	**0.34 (0.22, 0.55)**	**0.54 (0.37, 0.80)**	**0.53 (0.36, 0.79)**	**0.38 (0.24, 0.61)**	**0.59 (0.40, 0.88)**	0.69 (0.44, 1.07)
Level of education	0.92 (0.77, 1.09)	**0.71 (0.59, 0.87)**	**0.61 (0.45, 0.83)**	**0.69 (0.56, 0.85)**	**0.66 (0.56, 0.79)**	**0.64 (0.53, 0.77)**
SoC‐R	**0.41 (0.25, 0.67)**	**0.56 (0.38, 0.83)**	**0.33 (0.35, 0.79)**	**0.36 (0.21, 0.62)**	**0.47 (0.31, 0.72)**	**0.56 (0.34, 0.93)**

*Note*: All comparisons are with reference class *Online and offline helpers*. Bold indicates statistical significance.

** Odds ratios with 95% confidence limits that do not include 1 can be considered to reflect a significant group difference.

### 4.6 Associations between latent classes and perceptions of community resilience

Estimated means of the two community resilience outcome variables for each of the four latent classes are displayed in Table 4. The overall test of significance of differences among the classes using the Wald test was significant for the Transformative potential (*χ* ^*2*^ = 51.737, *p* < 0.001) and Disaster management CART subscales (*χ* ^*2*^ = 13.626, *p* < 0.01). The results of pairwise comparisons are reported in Table 7. In regard to the perceptions of community Transformative potential, the members of the *Online and offline helper* class reported greater scores than any other class, and *Money donors* and *Neighbour helpers* reported greater scores compared to *Online health information sharers*. The members of the latter class reported lower perceptions of community Disaster management than any other group.

**TABLE 7:** Results of pairwise comparisons of mean scores of perceptions of community resilience variables by class for the full sample

	Wald test χ^2^					
	1 vs. 2	1 vs. 3	1 vs. 4	2 vs. 3	2 vs. 4	3 vs. 4
Transformative potential	4.604***	9.435**	0.575	51.509***	13.308***	10.249**
Disaster management	0.822	6.015*	0.339	11.379**	1.638	4.323*

*Note*: 1 = Money donors, 2 = Online and offline helpers, 3 = Online health information sharers, 4 = Neighbour helpers.

* *p* < .05.

** *p* < .01.

*** *p* < .001.

## 5 DISCUSSION

This study used the person‐centred approach to examine the patterns of prosocial behaviours exhibited by Italians during the March–May 2020 COVID‐19 lockdown. The results provide insight on how prosociality is expressed under collective quarantine conditions when face‐to‐face activities are strongly limited.

Four classes that featured different patterns of behaviours were identified. Three profiles—representing 40% of the sample—were characterised by a single dominant behaviour (i.e., donating money, sharing verified health information online or helping a neighbour), whereas one group (i.e., *Online and offline helpers*) engaged in a variety of online and offline, low‐ and high‐cost altruistic conducts. The results demonstrate that a considerable proportion of people engaged in a single exclusive behaviour, but the majority expressed their prosociality in multiple ways. These results reflect the multidimensionality of the altruistic conduct, which includes helping, sharing, comforting, guiding, rescuing but also the degrees of effort involved in such conducts (Eagly, 2009; Marzana, Marta, & Pozzi, 2012; Penner, Dovidio, Piliavin, & Schroeder, 2005). Donating money or sharing information online requires a limited amount of effort and commitment (Sproull et al., 2013). For this reason, *Online and offline helpers* appear to be the most prosocial profile: individuals belonging to this class have engaged in various altruistic behaviours, some of which required a high level of personal effort and involvement.

Multigroup analyses demonstrated that the same (or very similar) behavioural patterns can be found in all age groups. Contrary to our expectations, we did not find young adults to be more active online than older adults. It is possible that older adults were forced by the circumstances to become rapidly familiar with online services (Xie et al., 2020). An alternative explanation is that the study recruitment strategy via an online survey reduced the participation of people less familiar with technology. In addition, even though the *Online and offline helper* class looked the same across age groups, people aged over 35 years were more likely than young adults to engage in most prosocial behaviours we measured. This result is likely to reflect older adults' greater disposition to help others particularly when the context is socioemotionally relevant as it is during emergencies (Eisenberg & Spinrad, 2014; Padilla‐Walker, Memmott‐Elison, & Nielson, 2018; Wray‐Lake, Schulenberg, Keyes, & Shubert, 2017). It is also possible that, under such exceptional circumstances, older adults felt more competent or have been deemed more competent by others, and therefore put themselves into play more than young adults (Beadle, Sheehan, Dahlben, & Gutchess, 2015). Further investigations are necessary to better understand age‐related prosocial behaviour under collective quarantine conditions.

The analyses demonstrated that being a man, having achieved a higher level of education and reporting higher SoC‐R scores were associated with belonging to the *Online and offline helper* class. This result is consistent with those of previous studies revealing the gendered nature of prosociality and its relation with socio‐cultural and economic (Cicognani et al., 2012; Malin et al., 2015; Wilson, 2000). Women have a propensity for more relational prosocial behaviour and for bonding with others in close and dyadic relationships (e.g., helping a neighbour). Conversely, men are likely to intervene under emergency circumstances when real or perceived physical risks are involved, and their action is more collectively oriented (Diekman & Clark, 2015; Eagly, 2009; Espinosa & Kovářík, 2015).

As expected, a greater SoC‐R was associated with engaging in a variety of altruistic behaviours (i.e., *Online and offline helpers*) to benefit the wider social and community context, compared to one‐off behaviours such as donating money. This is consistent with the results of research, indicating there is a positive relationship between community responsibility and community engagement (Prati et al., 2020; Procentese & Gatti, 2019; Procentese, Gatti, & Falanga, 2019).

Lastly, consistent with our expectations, *Online and offline helpers* perceived their community as more capable of coping with the emergency and reported the greatest perceptions of community Transformative potential resilience. We speculate this reflects their expectations that community members are in turn more prone to help others as they do (Procentese, De Carlo, & Gatti, 2019). Conversely, limiting their action to a single behaviour with little interaction with other community members, *Online health information sharers* felt their community was less resilient. These findings are in accordance with Jetten, Reicher, Haslam, and Cruwys (2020), who pointed out that ‘*resilience […] arises when people come together as a group, when they come to see others as a source of support*’ (p. 9).

Several limitations of the current study suggest avenues for future research. First, the cross‐sectional design constrains the interpretation of causal effects. Further longitudinal and mixed‐method research is needed to better examine causal relationships and get a deeper understanding of these issues (Aresi, Henderson, Hall‐Campbell, & Ogley‐Oliver, 2017). A second limitation is that the present study employed only self‐report measures, which might be susceptible to response bias. Third, as required by LCA, dichotomous items were used to establish a typology of patterns of prosocial behaviours. Future studies may attempt to investigate whether behaviours also cluster in terms of intensity in addition to type. Lastly, our analyses were not based on a representative sample. People aged 65 years and above and who did not engage in any prosocial behaviour were excluded, and the recruitment strategy via an online survey likely reduced the participation of people less familiar with technology. These issues limited our conclusions especially in regard to the correspondence between prosocial profile prevalence in our sample and the general adult population. Future studies with representative samples from all age groups in Italy and other countries are needed to confirm and generalise these findings.

## 6 CONCLUSIONS AND IMPLICATIONS FOR RESEARCH AND PRACTICE

The current study contributes to the literature by providing a typology of prosocial behaviours under collective quarantine conditions. Our findings can inform targeted interventions and communication campaigns to foster spontaneous altruism during foreseeable similar circumstances in the future. While it is beyond the scope of this study to critique whether some patterns of prosocial behaviours are preferred over others, programmes might be developed to eliminate barriers that hinder individuals from helping others in multiple ways. In order to create custom strategies for specific groups, future studies should investigate what factors explain why some individuals limit their action to a single gesture and encourage them to adopt a broader approach. Individuals can then be motivated to commit their time to provide practical and emotional support to others, thus generating a virtuous cycle pattern of reciprocity that can continue beyond the time of the health emergency (Erreygers, Vandebosch, Vranjes, Baillien, & De Witte, 2018). The results also indicate that individuals who engaged in a variety of online and offline prosocial behaviours displayed a greater, but still low, chance of working as volunteers. Community organisations may target this subgroup to recruit new people for the time of the emergency. In this regard, the literature on episodic volunteering may offer insight on how to best recruit and motivate volunteers under such specific circumstances (Pozzi, Meneghini, & Marta, 2019).

From a public health perspective, community engagement proved critical to contain epidemics in the past (Laverack & Manoncourt, 2015). Stakeholders and policymakers should consider the relevance of the direct involvement of community organisations and citizens as ‘lay actors’ of prosocial actions that can contribute to supporting people navigate through the harshness of the emergency and can be a valuable aid to public services. Finally, we suggest that our study makes an important methodological contribution to the field of prosocial behaviours. Our use of LCA allowed developing a multifaceted and thorough portrait of these behaviours during health emergencies. We recommend LCA as an important tool for future studies in this field.

## CONFLICT OF INTEREST

The authors declared no potential conflicts of interest with respect to the research, authorship and/or publication of this article.

## ETHICS STATEMENT

All aspects of this study were scrutinised and approved by the Università degli Studi di Bologna Institutional Review Board. The study was conducted in accordance with the Declaration of Helsinki.

## Supporting information


**Table S1.** Item‐response probabilities and class prevalence rates for the five‐class latent class analysis (LCA) model for the full sample.

**Data S1.** Supporting information.

## Data Availability

The data that support the findings of this study are available in the Università degli Studi di Bologna AMS Acta repository at http://doi.org/10.6092/unibo/amsacta/6634. Data will be made freely accessible on May the 31st 2022. In the meantime, the data are available upon reasonable request.
