# Supplementary material for: Prosocial behaviours under collective quarantine conditions. A latent class analysis study during the 2020 COVID‐19 lockdown in Italy
Source: J Community Appl Soc Psychol. 2021 Sep 18;32(3):490–506. doi: 10.1002/casp.2571 (PMC8653383; doi:10.1002/casp.2571)
Supplement: Supplementary file 1 — Table S1. Item‐response probabilities and class prevalence rates for the five‐class latent class analysis (LCA) model for the full sample. [file CASP-32-490-s002.docx]

**Table S1**. *Item-response probabilities and class prevalence rates for five-class LCA model for the full sample*.

|  | **Latent Class** | | | | |
| --- | --- | --- | --- | --- | --- |
|  |  |  |  |  |  |
|  | 1 | 2 | 3 | 4 | 5 |
| Volunteered | 0.12 | 0.04 | 0.08 | 0.17 | 0.05 |
| Donated money | 0.38 | **1.00** | 0.33 | 0.10 | 0.27 |
| Helped a neighbour | **0.62** | 0.00 | **1.00** | 0.19 | 0.35 |
| Shared competencies | 0.50 | 0.10 | 0.09 | 0.28 | 0.06 |
| Shared health advice | **0.83** | 0.13 | 0.05 | 0.00 | **1.00** |
| Helped school children | 0.31 | 0.08 | 0.08 | 0.22 | 0.04 |
| Created hope content online | **0.58** | 0.11 | 0.11 | 0.45 | 0.38 |
| Created an online sharing platform | 0.42 | 0.08 | 0.12 | 0.27 | 0.10 |
| **Estimated Prevalence** | 20.3% | 9.8% | 21.1% | 14.7% | 34.1% |
