# Supplementary material for: Prosocial behaviours under collective quarantine conditions. A latent class analysis study during the 2020 COVID‐19 lockdown in Italy
Source: J Community Appl Soc Psychol. 2021 Sep 18;32(3):490–506. doi: 10.1002/casp.2571 (PMC8653383; doi:10.1002/casp.2571)
Supplement: Supplementary file 2 — Data S1. Supporting information. [file CASP-32-490-s001.pdf]

Prosocial behaviours under collective quarantine conditions. A latent class analysis study during the 2020 COVID-19 lockdown in Italy

Aresi, G.*, Procentese, F.+, Gattino, S.#, Tzankova, I.°, Gatti, F.+, Compare, C.°, Marzana, D.*, Mannarini, T.§, Fedi, A.#, Marta, E.*, Guarino, A.°

**CERISVICO Research Centre for Community Development and Organisational quality of life, Università Cattolica del Sacro Cuore; + University of Naples Federico II; # University of Torino; ° University of Bologna; § University of Salento.*

Statement of relevance

- The study presented in this article examined patterns of prosocial behaviours exhibited by Italian adults during the March–May 2020 COVID-19 lockdown.
- Results offer insight on how prosociality is expressed under collective quarantine conditions when face-to-face activities are strongly limited.
- A four-type typology of patterns of prosocial behaviours was identified:
  - Money donors (7%), Online & offline helpers (59%), Online health information sharers (21%), and Neighbour helpers (13%).

Key results

- Our results reflect the multidimensionality of the altruistic conduct, but also the degrees of effort involved in such conducts (e.g., relatively little when just donating money or sharing information online).
- *Online & offline helpers* appear to be the most prosocial profile:
  - individuals belonging to this type have engaged in various altruistic behaviours and reported the highest sense of community responsibility and greatest perceptions of community resilience to emergency.

Implications for practice

- Our findings can inform targeted interventions and communication campaigns to foster spontaneous altruism during collective quarantine conditions.
- *Online & offline helpers* felt the most responsible for their community and were more likely to be involved in voluntary activity. Community organisations may target this subgroup to recruit new people for the time of the emergency.
- Individuals who limit their action to a single gesture, such as donating money, could be encouraged to adopt a broader approach.
- Barriers that hinder individuals from helping others in multiple ways should be eliminated as well.
